# Frequency of intraoperative cardiac arrest and medium-term survival

**DOI:** 10.1590/1516-3180.2013.1315507

**Published:** 2013-10-01

**Authors:** Ilana Sebbag, Maria José Carvalho Carmona, Maria Margarita Castro Gonzalez, Hermes Marcel Alcântara, Rolison Gustavo Bravo Lelis, Flavia de Oliveira Toledo, Gustavo Fábio Aranha, Rafael Ximenes do Prado Nuzzi, José Otávio Costa Auler

**Affiliations:** I MD. Attending Anesthesiologist, Instituto Central (IC), Hospital das Clínicas (HC), Faculdade de Medicina da Universidade de São Paulo (FMUSP), São Paulo, Brazil.; II MD, PhD. Assistant Professor, Discipline of Anesthesiology, Faculdade de Medicina da Universidade de São Paulo (FMUSP), São Paulo, Brazil.; III MD, PhD. Cardiologist, Instituto do Coração (InCOR), Hospital das Clínicas (HC), Faculdade de Medicina da Universidade de São Paulo (FMUSP), São Paulo, Brazil.; IV MD. Resident Physician in Psychiatry, Instituto de Psiquiatria (IPq), Hospital das Clínicas (HC), Faculdade de Medicina da Universidade de São Paulo (FMUSP), São Paulo, Brazil.; V MD. Attending Anesthesiologist, Instituto de Ortopedia (IOT), Hospital das Clínicas (HC), Faculdade de Medicina da Universidade de São Paulo (FMUSP), São Paulo, Brazil.; VI MD. Attending Anesthesiologist, Instituto do Câncer do Estado de São Paulo (ICESP), Hospital das Clínicas (HC), Faculdade de Medicina da Universidade de São Paulo (FMUSP), São Paulo, Brazil.; VII MD. Resident Physician in Anesthesiology, Instituto Central (IC), Hospital das Clínicas (HC), Faculdade de Medicina da Universidade de São Paulo (FMUSP), São Paulo, Brazil.; VIII MD, PhD. Head Professor of Discipline of Anesthesiology, Faculdade de Medicina da Universidade de São Paulo (FMUSP), São Paulo, Brazil.

**Keywords:** Intraoperative complications, Heart arrest, Cardiopulmonary resuscitation, Blood circulation, Survival rate, Complicações intra-operatórias, Parada cardíaca, Ressuscitação cardiopulmonar, Circulação sanguínea, Taxa de sobrevida

## Abstract

**CONTEXT AND OBJECTIVE:**

Although advances in surgical and anesthetic techniques have reduced perioperative morbidity-mortality, the survival rate following cardiac arrest remains low. The aim of this study was to evaluate, over the course of one year, the prevalence of intraoperative cardiac arrest and the 30-day survival rate after this event in a tertiary teaching hospital.

**DESIGN AND SETTING:**

Prospective cohort study in a tertiary teaching hospital.

**METHODS:**

Following approval by the institutional ethics committee, anesthetic procedures and cases of intraoperative cardiac arrest between January and December 2007 were evaluated. Patients undergoing cardiac surgery were excluded. The data were gathered prospectively using the modified Utstein model, with evaluation of demographic data, pre-arrest conditions, intraoperative care, care during arrest and postoperative outcome up to the 30th day. The data were recorded by the attending anesthesiologist.

**RESULTS:**

During the study period, 40,379 anesthetic procedures were performed, and 52 cases of intraoperative cardiac arrest occurred (frequency of 13:10,000). Among these, 69% presented spontaneous return of circulation after the initial arrest, and only 25% survived for 30 days after the event. The following factors were associated with shorter survival: American Society of Anesthesiologists physical status IV and V, emergency surgery, hemorrhagic events, hypovolemia as the cause of arrest and use of atropine during resuscitation.

**CONCLUSIONS:**

Although the frequency of cardiac arrest in the surgical environment has declined and resources to attend to this exist, the survival rate is low. Factors associated with worst prognosis are more frequent in critical patients.

## Abstract (pt)

**CONTEXTO E OBJETIVO:**

Apesar de avanços nas técnicas cirúrgicas e anestésicas terem reduzido a morbimortalidade perioperatória, a taxa de sobrevivência após parada cardíaca (PC) permanece baixa. O objetivo deste estudo foi avaliar, ao longo de um ano, a incidência de PC intraoperatória e de sobrevida por 30 dias após esse evento em um hospital terciário de ensino.

**TIPO DE ESTUDO E LOCAL:**

Estudo de coorte prospectivo em hospital terci ário de ensino.

**MÉTODOS:**

Após aprovação pela Comissão de Ética institucional, os procedimentos anestésicos e os casos de PC intraoperatórios foram avaliados no período de janeiro a dezembro de 2007, excluindo-se pacientes submetidos a cirurgia cardíaca. Os dados foram coletados prospectivamente utilizando o modelo Utstein modificado, com avaliação dos dados demográficos, das condições pré-PC, dos cuidados intraoperatórios e durante a PC e do desfecho pós-operatório até o trigésimo dia. Os dados foram registrados pelo anestesiologista assistente.

**RESULTADOS:**

Durante o per íodo de estudo, 40.379 procedimentos anestésicos foram realizados, com ocorrência de 52 casos de PC intraoperatória (frequência de 13:10000). Entre estes, 69% apresentaram retorno da circulação espontânea após a primeira parada e apenas 25% sobreviveram 30 dias após o evento. Os seguintes fatores foram associados com menor sobrevida: estado físico IV e V (Sociedade Americana de Anestesiologia), cirurgias de emergência, eventos hemorrágicos, hipovolemia como causa da parada e uso de atropina durante a reanimação.

**CONCLUSÕES:**

Embora a frequência de PC no ambiente cirúrgico tenha caído e existam recursos para seu atendimento, a taxa de sobrevivência é baixa. Fatores relacionados a pior prognóstico são mais frequentes em pacientes graves.

## INTRODUCTION

Despite great advances in intraoperative physiological monitoring and surgical anesthesia techniques, perioperative cardiopulmonary arrest (CPA) is still the most catastrophic complication during surgery and compromises the postoperative recovery of many patients. The mechanisms relating to intraoperative CPA differ from those responsible for out-of-hospital events. Hypovolemia due to bleeding is the most frequent cause of intraoperative CPA.^1^ The reported frequency of intraoperative cardiac arrest varies widely, from 2.56 to 39 cases per 10,000 operations.^1-6^ However, it is difficult to assess the quality of cardiopulmonary resuscitation (CPR) because of an absence of standardized templates for describing resuscitation efforts and for classifying the intraoperative complexity of such cases. Furthermore, limited data are available regarding the predictors of CPA survival and 30-day clinical outcomes.

In 1990, a group of CPR experts met at the International Resuscitation Conference in Utstein, Norway, and developed a set of guidelines, known as Utstein-style reporting, to standardize CPR reports.^7^ On the basis of these consensus guidelines, we developed a questionnaire to assess cases of intraoperative CPA.

### OBJECTIVE

The aim of this study was to evaluate the frequency and outcomes of intraoperative CPA cases over a one-year period in a tertiary teaching hospital, using Utstein-style reporting. 

### METHODS

Following approval by the institutional ethics committee for human research, the CPA frequency was evaluated in the operating rooms or diagnostic procedure rooms of all the units of the hospital.

### Design and location

This was a prospective single-center cohort study, in a tertiary teaching hospital. 

### Sample size:

A time-limited convenience sample was used, covering the period from January to December 2007 and totaling 40,379 anesthetic procedures.

### Inclusion criteria

Among all the patients who underwent surgical anesthetic procedures, including regional anesthesia alone, those who experienced CPA and were subjected to cardiopulmonary resuscitation maneuvers with external or internal thoracic compressions and/or those who underwent cardiac defibrillation were included in the study.

### Exclusion criteria

Patients who were undergoing cardiac surgery and those who did not receive CPR were excluded. In addition, "do not resuscitate" (DNR) cases were not included. The criteria for DNR were defined previously, in cases of palliative care undergoing minor surgeries for supportive procedures; or in the operating room, by the surgical team, in cases of failure of all life support efforts before the cardiac arrest.

### Intervention and outcome

Cardiac arrest was defined as the cessation of cardiac mechanical activity, as determined by the absence of a palpable central pulse. Patients experiencing CPA were treated using advanced cardiac life support (ACLS) maneuvers. Among the patients with more than one cardiac arrest, only the first arrest was analyzed. A data-gathering form, based on the Utstein model,^8,9^ was completed during and after each cardiac arrest by the attending anesthesiologist, together with one of the present authors. This form was used to collect information relating to the following: age; gender; preoperative clinical status, i.e. the American Society of Anesthesiologists (ASA) physical status, including the hemodynamic conditions, presence of infection and use of sedation and mechanical ventilation; types of anesthetic drugs used; type of surgery; clinical signs and symptoms before, during and after CPA; causes of CPA; interventions during CPR; and patient outcome. All patients were followed up on a daily basis until the 30^th^ day after the event or until the patient's death. 

For the primary aim of this study, the frequency of cardiac arrest was defined as the number of occurrences of this adverse event divided by the total number of anesthetic procedures performed in the hospital over the period considered. From the anesthesia records and the data-gathering form, the causes of cardiac arrest and death were retrospectively assigned by three of the present authors as one of the following: entirely related to anesthesia when anesthesia was the only or the major contributory factor; partially related to anesthesia when the patient's disease/condition or the surgical procedure was a contributory factor, but anesthesia represented an additional factor; entirely related to surgery; or entirely related to the patient's disease/condition.^10^ The cause of the event was deemed to have been established when there was a majority conclusion. There was some discussion in relation to a few cases. 

The outcome was evaluated in terms of the return of spontaneous circulation, 24-hour and 30-hour survival after the procedure, and the Glasgow Coma Scale score 30 days after surgery.

### Statistical analysis

Quantitative values were presented as means ± standard deviation (SD), and discontinuous values as percentages. Quantitative values were compared using the two-sided, unpaired Student's t test or Mann-Whitney U test for non-Gaussian distributions. Discontinuous variables were compared using chi-square tests or Fisher's exact test, as indicated. All data analyses were performed using the Statistical Package for the Social Sciences (SPSS) software (version 17.0, SPSS Inc., Chicago, United States) for Windows. Differences with P < 0.05 were considered statistically significant.

### RESULTS

Between January and December 2007, 40,379 anesthetic procedures were performed, and 52 cases of intraoperative cardiac arrests occurred (a frequency of 13:10,000). The frequency of CPA for each unit of the hospital is presented in Table 1. The CPA frequency was highest in the Central Institute, which is the site of the emergency and trauma unit, where it was 15:10,000 for emergency cases. This frequency was associated with shorter survival than in elective cases (P = 0.008). Excluding the emergency cases, the CPA frequency among the elective cases in the Central Institute was similar to that of the other units (5:10,000). The presence of hemorrhagic events before or during surgery (P = 0.024) also correlated with reduced survival. Overall, the incidence of deaths that occurred up to 30 days after CPA was 10 per 10,000 anesthetic procedures. 

**Table 1:** Frequency of cardiopulmonary arrest (CPA) during anesthetic procedures according to hospital unit

	Central Institute		Children's Institute (En/SC)		Psychiatric Institute (CC/ECT)		Orthopedic Institute		Radiology Institute		Total
	CPAs	Total	CPAs	Total	CPAs	Total	CPAs	Total	CPAs	Total	
January	3	1,984	2	335		300	1	521		186	3326
February	1	1,833		283		245		450		198	3009
March	5	2,282		365	1	349		598		189	3783
April	8	2,004		318		306		542		192	3362
May	6	2,080		354		377		590		207	3608
June	4	2,003		331		296		534		200	3364
July	10	2,182		394		314		552		199	3641
August	1	2,274		422		369		591		200	3856
September	1	1,945		335		312		486		188	3266
October	3	2,130		360		353		533		149	3525
November	3	1,803		321		271		467		127	2989
December	3	1,430		290		292		471		167	2650
Total	48	23,950	2	4,108	1	3,784	1	6,335	0	2,202	40,379
Frequency	20.04:10,000		4.86:10,000		2.64:10,000		1.57:10,000		0		12.87:10,000

Forty-eight cases of CPA occurred in adults (92%), and four cases occurred in children (8%). The distribution of CPA according to gender and age among adults and children is presented in Table 2, along with the patients' preexisting conditions. Among these, preoperative hypertension, heart failure and renal insufficiency, were the most prevalent ones. The more decompensated the basal disease or general clinical condition was, more frequently it led to decreased survival, as shown in Table 3 (P = 0.002 for ASA physical status IV and V, compared with ASA I, II and III). Patient-related diseases or conditions were the most frequent immediate causes of CPA (27 cases, 52%). In 21 CPA cases (40%), the anesthesia procedure was considered to be partially related to the adverse event; in 4 cases (8%), the CPA was considered to be entirely related to the surgery.

**Table 2:** Patients' characteristics before cardiac arrest

Demographic data	
Male gender, n (%)	32 (62)
Female gender, n (%)	20 (38)
Age of adult patients (years), mean ± SD	50 ± 19
Age of pediatric patients (years), mean ± SD	4 ± 4
**Preexisting conditions, n (%)**	
Renal insufficiency	12 (23)
Diabetes mellitus	9 (17)
Asthma/COPD	4 (8)
Hypertension	17 (33)
Heart failure	9 (17)
Hepatic insufficiency	4 (8)
Trauma	10 (19)
Oncological diseases	5 (10)
Acute hemorrhage	12 (23)

**Table 3:** American Society of Anesthesiologists (ASA) physical status classification and type of surgery. Numbers of patients and survival at 30 days, n (%)

ASA physical status classification	n (%)		Survival at 30 days	
			n (%)	P
ASA I	4 (8)	32	13 (40)	0.002	32	13 (40)
ASA II	8 (15)					
ASA III	20 (39)					
ASA IV	12 (23)	20	0 (0)			
ASA V	8 (15)					
Type of surgery	Emergency	37 (71)	5 (14)	0.008
	Elective	15 (29)	8 (53)	

Cardiac arrest was less common during procedures using regional anesthesia alone and in diagnostic procedures (Table 4). The majority of the events occurred during the maintenance period of general or combined anesthesia. At the time of CPA, all the patients were under electrocardiogram (EKG) monitoring and had at least one vascular access (Table 5).

**Table 4:** Characteristics of the surgical and anesthetic procedures

	n (%)
Type of anesthesia	
General	47 (90)
Regional	2 (4)
Combined	3 (6)
**Types of surgical procedure**	
Thoracic	10 (19)
Abdominal nonvascular	14 (27)
Neurosurgical	8 (15)
Orthopedic	7 (14)
Vascular	6 (12)
Diagnostic procedures	3 (6)
Others	6 (12)
**Stage of anesthesia**	
Induction	4 (8)
Maintenance	45 (87)
Recovery	4 (8)

**Table 5:** Interventions in place before the arrest

	n (%)
Vascular access	52 (100)
Electrocardiographic monitor	52 (100)
Pulse-oximeter	52 (100)
Noninvasive blood pressure measurement	42 (81)
Invasive blood pressure measurement	19 (37)
Capnography	42 (81)
Central venous access	19 (37)
Pulmonary artery catheter	1 (2)

As presented in Table 6, the most frequent first-documented arrest rhythm was pulseless electrical activity (28 cases, 54%). The most common immediate cause of the adverse event was hypovolemia (42%), followed by respiratory (21%) and metabolic (21%) disturbances. Hypovolemia and respiratory disturbances were associated with reduced survival (P = 0.028 and P = 0.026, respectively). Table 6 also indicates that pharmacological interventions during CPR and administration of atropine during resuscitation efforts were correlated with shorter survival (P = 0.022). Overall, 36 patients (69%) experienced return of spontaneous circulation (ROSC), but only 25% survived for 30 days after the event. Twenty patients (39%) were alive 24 hours after the arrest, but only 13 patients survived for 30 days after the event (25%). Out of the patients who survived for 30 days, only six (46%) had Glasgow Coma Scale scores of 14 or 15.

**Table 6:** Characteristics of the cardiac arrest

	n (%)
First pulseless rhythm	
Ventricular tachycardia or ventricular fibrillation	6 (12)
Pulseless electrical activity	28 (54)
Asystole	7 (14)
Bradycardia evolving to asystole	11 (21)
**Immediate causes of the event**	
Hypovolemia	22 (42)
Respiratory cause: inadequate ventilation, inadequate oxygenation and others	11 (21)
Myocardial ischemia	7 (14)
Metabolic/electrolyte disturbances	11 (21)
Pulmonary embolus	2 (4)
Pneumothorax	1 (2)
Arrhythmia	4 (8)
Cardiac tamponade	1 (2)
Myocardial depression	4 (8)
Septic shock	4 (8)
Unknown	3 (6)
**Pharmacological interventions**	
Epinephrine	50 (96)
Epinephrine > 3 doses	30 (58)
Atropine	31 (60)
Calcium chloride/gluconate	16 (31)
Sodium bicarbonate	15 (29)
Fluids	33 (64)
Amiodarone	3 (6)

### DISCUSSION

The results from this prospective study confirmed that intraoperative CPA is related to high mortality. The highest incidence of CPA occurred among ASA physical status IV and V patients,^5^ and during emergency procedures. The combination of these two factors may increase the risk of the event.

The frequency of and mortality due to CPA observed in the present study were lower than those found in another Brazilian study by Braz et al. published in 2006,^10^ which reported a CPA-toprocedure ratio of 35:10,000. The difference between these results may be explained by the large number of anesthesia procedures performed for diagnostic purposes and the inclusion of postanesthetic cases in that study.^10^ Although we constantly monitored for events and carefully examined them, we cannot completely deny the possibility that unreported events might have occurred in our hospital, which could have led to underestimation of the number of events. We already keep constantly in touch with the surgical teams in order to avoid this possibility, but creation of an institutional database might make our counting even more trustworthy. 

Intraoperative cardiac arrest and mortality can be used as a measurement of the quality and safety of anesthesia. However, it is often difficult to define the relationship between this adverse event and anesthesia. According to the classifications used in this study,^10^ the majority of the cases were considered to be completely related to the patient's condition or only partially related to the anesthesia. Cardiac arrest attributable to anesthesia occurs in 0.5 to 1.0 case per 10,000 interventions,^5,11^ with higher incidence in pediatric cases (1-5 per 10,000),^11^ but also higher survival rates.5 This information suggests that the patient's age, preexisting diseases or traumas and new surgi-cal interventions play important roles in the risk of CPA. The results from the present study did not indicate any association between age and outcome.

Understanding "anesthesia-related/caused" mortality remains challenging.^12^ Increasing the awareness of the rate of perioperative mortality, and specifically anesthesia-related mortality, may improve the ability to predict which patients are at increased risk of cardiac arrest and to provide the management necessary to prevent this adverse event or to improve the outcome.^13^ In addition to technological improvements, it is important to emphasize the value of continuous training and to update medical teams according to recent guidelines.^11^

Although we did not use any device to evaluate the quality of the CPR performed in this study, no harm to any patients was reported to have been caused by this technique. In all cases, CPA was promptly diagnosed by the attending team, which included at least one well-trained anesthesiologist. Adequate measures for treating cardiac arrests were initiated quickly, in accordance with current protocols. In 1986, another study in the same institution reported that the incidence of CPA was 39 per 10,000 anesthetic procedures.^2^ Those authors also excluded cardiac surgery patients, thereby eliminating a possibly confounding variable. Many factors may have contributed to the decrease in intraoperative CPA frequency over this 20-year period, such as evolutions in monitoring and ventilation technologies, routine preoperative evaluation of elective patients, safer anesthetic drugs and, most importantly, better understanding of physiopathology, treatment of shock and training in advanced cardiac life support. Considering the high mortality attributed to CPA, great efforts should be invested in preventing this adverse event, especially in cases involving emergency situations and patients with hemorrhage, hypovolemia and respiratory complications, along with metabolic/electrolytic disturbances during anesthesia. 

Use of atropine during resuscitation efforts was associated with higher mortality in our study. The available evidence suggests that routine use of atropine during cardiac arrest treatment is unlikely to provide a therapeutic benefit.^14^ Recently, atropine was removed from the cardiac arrest algorithm, in the 2010 American Heart Association Guidelines for Cardiopulmonary Resuscitation and Emergency Cardiovascular Care.^15^

We observed that although return of spontaneous circulation occurred frequently, it was not related to a better outcome, considering that only 36.1% of those patients achieved 30-day survival. The high incidence of pulseless electrical activity could be related to hypovolemia, which was the most frequent cause of CPA in this study.

The poor neurological outcomes observed in this study provide corroboration that there is a need to improve postarrest care, as emphasized in the 2010 American Heart Association Guidelines.^15^

### CONCLUSIONS

Our findings indicate that the frequency of intraoperative cardiac arrest is decreasing and are in accordance with previous data. Additionally, intraoperative cardiac arrest was seen to be related to high mortality, especially in patients with previous comorbidities, emergency procedures, hypovolemia, respiratory complications and metabolic/electrolytic disturbances.
